# Structural alphabets derived from attractors in conformational space

**DOI:** 10.1186/1471-2105-11-97

**Published:** 2010-02-20

**Authors:** Alessandro Pandini, Arianna Fornili, Jens Kleinjung

**Affiliations:** 1 Division of Mathematical Biology, MRC National Institute for Medical Research, The Ridgeway, London NW7 1AA, UK; 2 Randall Division of Cell and Molecular Biophysics, King's College London, New Hunt's House, London SE1 1UL, UK

## Abstract

**Background:**

The hierarchical and partially redundant nature of protein structures justifies the definition of frequently occurring conformations of short fragments as 'states'. Collections of selected representatives for these states define Structural Alphabets, describing the most typical local conformations within protein structures. These alphabets form a bridge between the string-oriented methods of sequence analysis and the coordinate-oriented methods of protein structure analysis.

**Results:**

A Structural Alphabet has been derived by clustering all four-residue fragments of a high-resolution subset of the protein data bank and extracting the high-density states as representative conformational states. Each fragment is uniquely defined by a set of three independent angles corresponding to its degrees of freedom, capturing in simple and intuitive terms the properties of the conformational space. The fragments of the Structural Alphabet are equivalent to the conformational attractors and therefore yield a most informative encoding of proteins. Proteins can be reconstructed within the experimental uncertainty in structure determination and ensembles of structures can be encoded with accuracy and robustness.

**Conclusions:**

The density-based Structural Alphabet provides a novel tool to describe local conformations and it is specifically suitable for application in studies of protein dynamics.

## Background

Most proteins have arisen by natural selection to adopt a hierarchical three-dimensional fold, where regularly shaped structural motifs are packed together and form a hydrophobic core. The first description of two of these motifs introduced the concept of secondary structures (*α*-helix and *β*-sheet) and demonstrated that some local structures have a repetitive nature [1]. Later it was discovered that almost all regions of a protein backbone can be rebuilt by few substructures common to different proteins [2]. With increasing availability of high quality structures, it also became clear that some of the adopted conformations are realised much more frequently than others and more recently a detailed analysis of the Ramachandran space [3] for structures of different crystallographic resolution showed clustering of both secondary structure types and random coil conformations at distinct conformational attractors [4]. These attractors can be labelled as conformational 'states' and the protein structure can be considered as a sequence of conformational states. Indeed, classical secondary structure attribution encodes a protein structure into a sequence of states.

However, the protein fold cannot be fully reconstructed from the secondary structure sequence alone, because this code describes the conformation of single residues and provides too few states to capture the entire variety of local conformations. To overcome these limitations, comprehensive libraries of frequently occurring fragments spanning several, typically 4-7, residues were derived [5-12]. These libraries provide a richer choice of conformational states and they comprise intrinsically the structural correlation between consecutive residues. Using fragments, protein fold reconstruction can be achieved by superimposing chains of fragments in a head-to-tail arrangement.

Structural Alphabets are fragment libraries composed of a relatively small number of fragments that complement each other to form a 'universal code' of local conformations. Several Structural Alphabets have been derived [13] using methods such as cluster analysis [6,8,10,12,14], Kohonen maps [9] and Hidden Markov Models [11,15]. Generally machine learning strategies yielded better performing alphabets at the price of an indirect description of the conformational space. Despite the relative novelty, the potential of Structural Alphabets has been exploited for decoy generation [16], local structure prediction [9,17,18], sequence-based structural comparison [19], combined sequence-structure alignments [20], 3D structure alignment [21], structure mining [12,22-25], structure reconstruction from *C*^*α *^[26], fold classification [27], fold prediction [28], structure generation [29], *de novo *prediction [30,31], *de novo *backbone design [32], but not yet for molecular motions and conformational transitions. Therefore, a description of high quality Structural Alphabets is needed that allows for a projection of these properties into the conformational space of the alphabet, which would facilitate the development of applications that combine a static and dynamic description of proteins.

In this paper we devise a simple and explicit description of four-residue long fragments, the conformation of each being defined by three internal angles. All protein fragments were mapped as points in a three-dimensional space of these internal angles. Structural Alphabets were extracted directly from the conformational attractors on that fragment map and assessed in terms of their accuracy in reconstructing protein structures. A performance comparison was made with other Structural Alphabets of four-residue fragments. Finally the suitability of our best performing alphabet for the description of protein dynamics was assessed by encoding the different structures in a test set of conformation ensembles and measuring the correlation between local flexibility and encoding variability.

## Methods

### Dataset

A reference set of high quality protein structures was selected from ASTRAL SCOP 10 (v1.73), which includes domains with less than 10% sequence identity [33,34]. The degree of quality was measured by the Summary PDB ASTRAL Check Index (SPACI) [34]. This index provides information on the reliability and precision of protein structures. It includes three contributions: the quality of the experimental data (resolution), the quality of the fitting procedure (R-factor), and the quality of the deposited model structure (stereochemical accuracy). Only X-ray structures with complete backbone chains and SPACI quality scores > 0.5 were included, yielding a total of 1830 protein domains. The list of SCOP ids is available for download at http://mathbio.nimr.mrc.ac.uk/download/MK.dataset.txt.

**Figure 1:**
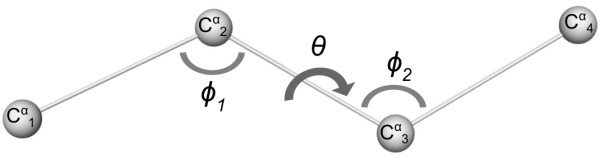
**Fragment definition**. *C*^*α *^atoms are represented as spheres. The conformation is entirely described by two pseudo bond angles (*ϕ*_1_, *ϕ*_2_) and one pseudo torsion angle (*θ*).

The dataset of local structures was defined as the collection of all four-residue long fragments within the reference set. A fragment is represented by the *C*^*α *^atoms of four consecutive residues. To avoid sampling bias and to allow for reconstruction, the extracted fragments may overlap in the source structure and neighbouring fragments share three atoms. For each fragment, three pseudo-angles were defined between their constituting *C*^*α *^atoms: the angle between atoms 1-2-3 (*ϕ*_1_), atoms 2-3-4 (*ϕ*_2_) and the torsion angle formed by atoms 1-2-3-4 (*θ*) (see Figure 1). Angle values were computed *via *the Cartesian coordinates of the *C*^*α *^atoms. Note that these angles are not directly related to the Ramachandran angles (Φ, Ψ), as our fragment definition embodies a coarse grained representation of the backbone without consideration of the peptide bond geometry.

A molecule composed of *N *atoms possesses 3*N *degrees of freedom. Removal of trivial rigid body motions and bond constraints yields 3 * 4 - 6 - 3 = 3 degrees of freedom, which are entirely described by the three independent internal angles *ϕ*_1_, *ϕ*_2 _and *θ*. Advantages of this representation are the conceptual simplicity, the ease of visualisation and the fast comparison of fragment geometries by angle differences instead of atom super-positioning.

### Density-based cluster analysis

Cluster analysis of the fragments extracted from the selected set of 1830 protein domains (325923 fragments) was performed in the conformational space of the three internal fragment angles (*ϕ*_1_, *ϕ*_2_, *θ*). The values of these angles were neither normalised nor standardised to preserve the original ratio between the variance in the torsional angle *θ *compared to the planar angles *ϕ*. This information is central to correctly detect the geometries associated with secondary structures and the transitions between them. A cubic grid with 2° resolution was defined to obtain an initial fragment density estimate. To reduce the computational complexity, irrelevant data were removed by an initial filtering step: those fragments in cubes containing in total less than 10 fragments were removed. The final dataset included 133254 fragments.

Extraction of a Structural Alphabet from the data cloud in this conformational space requires the identification of representatives within the high-density regions of point clusters. However, the data did not lend themselves to standard clustering methods, because of the large variation of cluster densities and the partial overlap of clusters with different densities. To overcome this problem, the OPTICS (Ordering Points To Investigate the Clustering [35]) method was implemented in C and applied to the dataset. A flow chart of the algorithm is given in Additional File 1: Supplementary Figure S1. OPTICS is based on a nearest neighbour walk through the data space, thereby ordering and recording pairwise point distances [36]. The approach is particularly suitable for the extraction of a Structural Alphabet, because fragments can be clustered hierarchically by density and representatives may be selected amidst the highest point densities. The algorithm requires only two input parameters: a neighbourhood radius (*ε*) and a minimum number of neighbour points (*MinPts*). We set *ε *to 200°; since the largest angular RMSD in the dataset is 78°, each point is reachable and this parameter had no influence on the results.

Briefly, the algorithm starts at a random data point, calculates the distance to all points within the neighbourhood radius (*ε*) and, if at least a minimal number of points (*MinPts*) is encountered, it records the nearest neighbour distance (Reachability Distance) and the smallest radius that encircles *MinPts *objects (Core Distance). If less than *MinPts *points fall within *ε*, the point is considered as noise. The algorithm repeats the same procedure for the nearest neighbour point and proceeds iteratively until all data points have been visited, thereby generating an ordered list. Our specific choice of *ε *implies that none of the fragments is labelled 'noise' and each is included in one cluster at least. This choice allows to scan afterwards for clusters at any density. Distances *d*_*ij *_were calculated as the root mean square deviation in angular coordinates (aRMSD) between fragment pairs. Angle differences of *ϕ*_1 _and *ϕ*_2 _naturally fell into the value range [0,180], while for *θ *periodicity was removed to retain the value range [0,180]. The ordered list of Reachability Distances (RDs) can be drawn as a comprehensive nearest neighbour distance plot (called Reachability Plot).

### Structural Alphabet extraction

The cluster structure emerges directly from the Reachability Plot. A variant of the Drop-Down algorithm [37] was implemented to extract the clusters and their representatives. The idea behind this algorithm is to hierarchically extract the clusters by progressively increasing a density threshold. Given a density threshold value, a cluster is defined by a contiguous series of (ordered) points having a density above the threshold. The algorithm works as follows:

1. Generate a list in which the fragments are sorted by decreasing Reachability Distance (increasing density) with 'merge sort'.

2. Parse the sorted list to find two fragments that are more than *MinPts *apart in the Reachability Plot; these fragments enclose a candidate cluster.

3. If the size of the candidate cluster is at least *MinPts *smaller than the parent cluster, label the candidate cluster as accepted cluster and remove all its points from the sorted list.

4. Repeat 2 until reaching the end of the sorted list.

In the first iteration, the entire Reachability Plot is scanned for root clusters; in following iterations, new clusters are extracted by processing the clusters that were identified at the previous step. The fragment with the lowest Core Distance (highest density of fragments in its neighbourhood) is taken as the representative of a cluster.

### Redundancy removal

As previously reported [8], the distribution of pairwise Euclidean distances between short protein fragments of fixed length is multi-modal, with one peak corresponding to intra-cluster distances (same fragment conformation) and the others to inter-cluster distances (different fragment conformation). Using the same principle, we derived a cutoff distance to remove redundant representatives from our set.

The distribution of pairwise distances was calculated for a random sample of 13326 fragments from the dataset. The Euclidean distance between the *C*^*α *^atoms after optimal superposition of all fragment pairs was computed. The resulting distance distribution of the intra-cluster peak is log-normal for values smaller than 1.0 Å and it was fitted using the maximum-likelihood method of the R [38] package MASS [39].

Occurrences within one standard deviation (0.307 Å) account for 84% of the data and 0.307 Å was selected as the redundancy distance cutoff for cluster representatives.

### Assessment by fit quality

The quality of a Structural Alphabet is generally assessed by its accuracy in approximating real protein structures. For this purpose the performance measure previously used by other authors was adopted: local fit and global fit accuracy [6]. A local fit is obtained if each position of a given protein structure template is overlaid with the best-fitting alphabet fragment. The coordinate root mean square deviation (cRMSD) on the position of each *C*^*α *^atom from the template protein is then calculated. A global fit is obtained if the best-fitting sequence of fragments is given and the protein structure is reconstructed by progressively overlaying the ends of neighbouring fragments in the sequence. In this case the cRMSD value is calculated after aligning the reconstructed structure with the template.

In both cases the median of the protein RMSD fit was calculated for a test set including 798 proteins from ASTRAL SCOP superfamily level with SPACI scores >0.5 [33,34]. Dataset and test set did not overlap. The list of SCOP ids of the test set is available for download at http://mathbio.nimr.mrc.ac.uk/download/MK.testset.txt.

For this analysis the global fit procedure described by [6] was implemented in C. Whenever possible, RMSD calculations were performed with Theobald's fast quaternion method [40]. A heap size of 2000 was used to ensure convergence of the encoding.

### Assessment by information content

The performance of a discrete state model (such as the Structural Alphabet) is a trade-off between the complexity invoked by its number of states and the ability to describe reality as reflected by its fit quality. A suitable metric for this trade-off is Akaike's Information Criterion (AIC), an entropy based measure of the goodness of fit for a given model with a varying number of parameters [41,42]. Being grounded in Information Theory, it provides a relative measure of the information loss by using the model instead of the real data. The general expression for a model with *k *parameters and a maximum likelihood *L *(of the fit) reads:(1)

If the model errors are normally and independently distributed, the AIC can be expressed in terms of the *n *residual errors of the fit (*ε*_*i*_) as(2)

The Structural Alphabets obtained for different values of *MinPts *were ranked according to their AIC. The residual errors were calculated in the form of a local fit RMSD of the 325923 fragments in the dataset. The Structural Alphabet with the lowest AIC was selected as the most informative alphabet, providing the highest fit performance for the lowest number of constituting fragments.

### Genetic Algorithm optimisation of combined fragments

To overcome any potential bias by the hierarchical cluster extraction, all fragments of the alphabets obtained with *MinPts *parameters in the range [10, 99] (initially 1709 representative fragments, reduced to 106 non-redundant fragments using a 0.1 Å cRMSD cut-off) were submitted to a global optimisation within the framework of a Genetic Algorithm [43]. The purpose of this optimisation is an independent alphabet derivation to verify that any potential methodological biases of the described OPTICS selection do not interfere with the performance of the selected alphabets.

The target size of the optimised Structural Alphabet was 25 fragments to match the size of our best performing alphabet (see below). Each fragment was represented by a gene in the form of a binary number, where [1/0] indicates either inclusion or exclusion with respect to the final subset. The fitness of the genome was calculated as the average local fit on the 10 top quality proteins (according to their SPACI score) in ASTRAL SCOP 40, which cover a diverse set of folds. The GA was run three times with a population size of 5000 genomes over 50 generations, cross-over breeding of the fittest 5% genomes and elitism (fittest genomes survive). The algorithm converged to a unique solution in 17 generations. The optimised subset was assessed by local fit, global fit and AIC.

### Analysis of native contacts and intrinsic flexibility

The local and global fit accuracy are robust and general measures of reconstruction quality. For applications other than reconstruction, specific quality measures should complement the assessment. The Structural Alphabets introduced here are intended to also capture the intrinsic flexibility of protein structures. This implies that the network of interactions in the native structure is correctly described in the reconstructed structures. Therefore, reconstructed structures were assessed in terms of their dynamics and residue interactions. A useful approach to test this is provided by the Gaussian Network Model (GNM) [44-46]. With this simple but elegant model it is possible to calculate the protein contact map and to derive an estimate of the Root Mean Square Fluctuation (RMSF) of the atom positions directly from a single structure.

In the GNM a protein of N residues is described by an elastic network where residues become nodes linked by harmonic springs. Each node is subjected to Gaussian fluctuations around its equilibrium position, defined by the coordinates of the *C*^*α *^atoms in the protein structure. The model is isotropic and has N degrees of freedom describing the amplitude of the fluctuation of each node. The force constant of the spring (*γ*) is generic and identical for each residue type. The associated inter-residue interaction potential is:(3)

where Δ*R *is an N-dimensional vector whose *i*^*th *^element is the fluctuation vector Δ*R*_*i *_of the individual residue *i*^*th*^, and Γ is the *N *× *N *Kirchhoff contact matrix with elements(4)

where *d*_*ij *_is the distance between residues *i *and *j *and the cutoff distance *r*_*C *_is 7 Å. The cross-correlation of fluctuations between residues *i *and *j *can be calculated from(5)

where *k*_*B *_is the Boltzmann constant and T is the absolute temperature. Accordingly, the mean-square fluctuations of the *C*^*α *^atoms can be extracted from the diagonal elements of Γ^-1^(6)

Estimated RMSFs and cross-correlation matrices were analysed for 798 pairs of native and reconstructed proteins from ASTRAL SCOP superfamily level with SPACI scores >0.5 (see above). The agreement of the RMSF profiles was measured by the Pearson correlation coefficient, while the similarity of the cross-correlation matrices was calculated by matrix overlap [47]:(7)

where A and B are the matrices to compare, *tr *is the trace operator and *d*(*A, B*) is the matrix difference:(8)

The overlap ranges from 0 (no overlap) to 1 (identical matrices).

### Analysis of conformational states in structural ensembles

The suitability of the proposed Structural Alphabet to analyse protein dynamics was further tested by investigating both the robustness of the fragments to small fluctuations and their ability to describe conformational transitions. To limit the computational effort, the analysis was performed on a set of 24 proteins. The conformational space of each protein was explored with the tCONCOORD method [48-50] that provides a more accurate model than GNM, since an all-atom representation of the system is used and anharmonicities in atom motions are allowed, but it is still simpler and faster than Molecular Dynamics (MD) simulations.

In tCONCOORD, ensembles of structures are generated by fulfilling a set of distance constraints between atom pairs. The permitted distance intervals are determined on the basis of the distance values found in the starting structure and of the type of the interaction (e.g. covalent bonds, hydrogen bonds, salt bridges or hydrophobic interactions), so that lower tolerances are used to describe stronger interactions. All the contacts in the original structure are preserved, except for 'under-wrapped' hydrogen bonds [49,51] which are considered unstable since they are not sufficiently shielded from the environment by hydrophobic groups. It has been shown that the detection of unprotected hydrogen bonds, together with the calibration of the distance constraint definition, allows the prediction of conformational transitions [49]. Moreover, even if the molecule description is less accurate than that provided by the force fields generally used in MD simulations and there is no explicit representation of the solvent, the collective motions and the overall RMSF profiles extracted from tCONCOORD ensembles have been found in good agreement with both MD and experimental results [48,49,52,53].

The test set of 24 proteins was extracted from a larger dataset of proteins annotated in the PiSite database [54] and currently used by the authors to study the role of flexibility in protein-protein interactions. To avoid the introduction of biases due to the over-representation of some secondary structure types or of some folds, the first four classes of SCOP (*α*, *β*, *α*/*β*, and *α *+ *β*) were equally represented and a given fold was considered only once (see Table 1). Moreover, within each group of six proteins belonging to the same SCOP class, it was ensured that the distributions of the total number of residues and of the ratio between structured (H, G, I, E and B in the DSSP [55] dictionary) and unstructured (T, S and unassigned) regions was covering a wide range (see Table 1). After full protonation and energy-minimisation with the GROMACS 3.3.3 package [56] and the OPLS-AA force field [57], tCONCOORD ensembles of 500 structures were generated for each protein.

**Table 1:** Dataset for the test on accuracy and robustness in encoding structural ensembles.

PDB ID	Chain	SCOP superfamily classification	H+E/L	size
1a4p	A	a.39.1	3.18	92
2dn2	C	a.1.1	2.92	141
2ilk	A	a.26.1	2.16	155
1v74	B	a.24.20	1.81	87
2hue	C	a.22.1	1.34	82
1mz4	A	a.3.1	1.30	131
1z5y	D	b.1.17	2.11	118
1n9r	G	b.38.1	2.09	68
2f3g	B	b.84.3	1.21	150
2z6k	D	b.40.4	1.09	117
2rac	A	b.6.1	0.91	105
1beh	B	b.17.1	0.69	183
1ay7	B	c.9.1	2.30	89
1qjc	B	c.26.1	1.85	157
2vrw	A	c.37.1	1.49	177
2d1p	B	c.114.1	1.43	119
2trx	B	c.47.1	1.30	108
1uex	C	c.62.1	1.22	202
1gy6	B	d.17.4	3.10	123
1oo0	A	d.232.1	2.06	144
1gd0	C	d.80.1	1.74	118
3eze	B	d.94.1	1.58	85
2uyz	A	d.20.1	1.20	156
2inc	C	d.15.12	1.07	83

H+E/L: ratio between regular (H+E) and irregular (L) secondary structure elements according to DSSP [55] (H+E = H+G+I+E and L = T+S+unassigned in the DSSP dictionary); size: number of residues.

A 'per-fragment' flexibility profile was obtained for each protein by calculating the RMSFs of *C*^*α *^over N-3 sliding windows of 4 residues. The roto-translational motion was eliminated by least-square superposition of the fragment in each frame to the reference starting structure. The value assigned to each window was calculated as the quadratic mean of the RMSF values of each *C*^*α *^in the fragment.

For comparison, for each protein the structures in the ensemble were encoded into structural strings by both local and global fit procedure as previously described (see "Assessment by fit quality"). Conversely to the encoding of a single structure, in the ensemble a given fragment position can be generally described by different letters. The letter variability per fragment was evaluated through the Shannon Entropy [58]:(9)

where *p*_*ij *_is the fraction of structures where fragment *i *was encoded by letter *j *and *k *is the total number of letters in the alphabet.

## Results

### Structural Alphabets from conformational attractors

**Figure 2:**
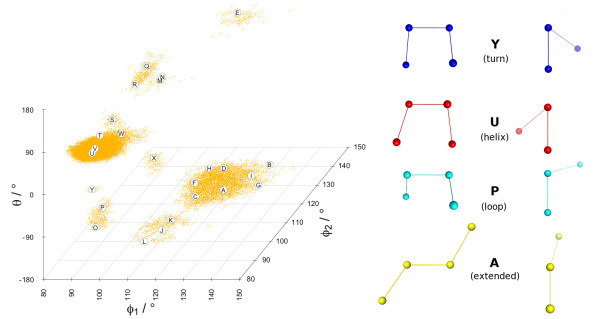
**Projection of the data into the conformational space of the internal angles (*ϕ*_1_, *ϕ*_2_, *θ*)**. Each dot (orange) corresponds to a fragment. The plot is split at the periodic boundary -180/180° of the *θ *angle, while the angle range of the *θ*_1 _and *θ*_2 _dimensions has been cropped to the populated region. Fragments of the M32K25 alphabet (see text) are shown as labelled circles and four selected fragments (Y blue, U red, P cyan, A yellow) are rendered as ball-and-stick models on the right panel. The left models illustrate the [*ϕ*_1_, *ϕ*_2_] angles and the right models the *θ *angle. The relation between the two views is a 90° rotation around a vertical axis in the paper plane and an adjustment to align the two central atoms to a Newman projection. Atom '1' is positioned left (left models) and front (right models). The plot was produced with the R package scatterplot3d [63] and the side panel with PyMol [64].

A plot of the conformational space of 133254 fragments derived from a set of 1830 high-resolution structures sampled from sequence-unrelated protein domains (less than 10% identity) is shown in Figure 2. The overall clustering of the fragments at conformational attractors is clearly visible. The existence and location of the high density regions around these attractors is an intrinsic property of the conformational space and it is within the tested limits independent of the dataset size and the resolution of the contained structures (see Additional File 1: Supplementary Figure S2 for details). One can readily identify main clusters around fragment conformations typically found in protein structures: helix, turn, extended and loop, as illustrated by the ball-and-stick models of the representatives of these clusters on the right panel. Although the main fragment clusters are already informative, their fine structure needs to be resolved computationally to extract fragment alphabets with a high information content (entropy).

**Figure 3:**
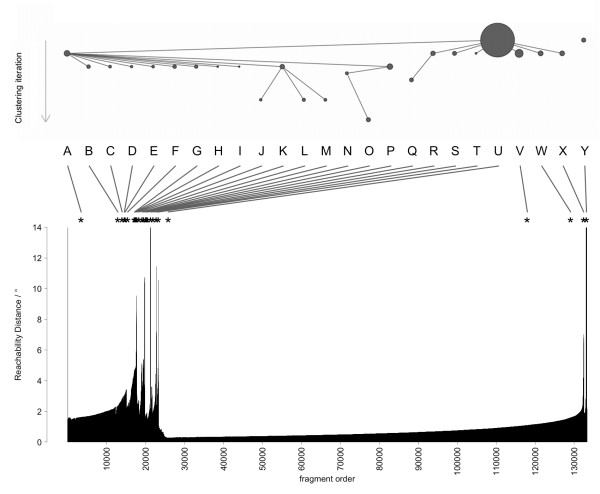
**Reachability Plot and clustering scheme for the alphabet M32K25**. The bottom panel shows the Reachability Distance (neighbour distance) of all fragments in the order of the nearest-neighbour walk. Short distances correspond in general to high cluster density. The Reachability Distance scale is cropped to 0-14° to preserve details. The order of cluster extraction is illustrated in the top scheme, where each circle represents a cluster, its size inversely proportional to its Core Distance. The labels in the middle panel are those of the resulting Structural Alphabet; lines indicate the location of the cluster representative in the Reachability Plot. For each cluster (dent region in the plot) the corresponding representative was selected by lowest Core Distance (top scheme).

Using the density clustering method OPTICS [35], a comprehensive and sorted density plot (Reachability Plot) of all fragments was obtained (see bottom panel Figure 3 for *MinPts *= 32 as example). The point density is approximated by a nearest-neighbour distance (Reachability Distance), measured as a Euclidean distance in the internal angle space (aRMSD) of the fragments' degrees of freedom (*ϕ*_1_, *ϕ*_2_, *θ*). The Reachability Plot shows the fine structure of the OPTICS data clustering.

This density plot was then processed with a variant of the Drop-Down algorithm [37], in which a density threshold was progressively increased, to extract the cluster structure (see Methods). A diagram of the hierarchical extraction is presented in the top panel of Figure 3, the resulting fragments and their location in the Reachability Plot are shown in the middle panel. In this scheme, frequently occurring fragments are selected first, rarer conformations later, which can also be interpreted as the importance of the attractor (cluster) in the conformational space. The collection of extracted fragments forms the Structural Alphabet. The rugged fine structure of the data density combined with the sensitive clustering method yielded some representatives with near-identical conformations. A plot of all pairwise fragment distances (cRMSD) in the dataset showed that the intra-cluster peak follows a log-normal distribution; fragments were deemed redundant and removed if their distance to an accepted representative within the intra-cluster peak was shorter than a cutoff value (0.307 Å, see Methods). An example of the distribution of fragments of a Structural Alphabet is shown in Figure 2; each fragment is indicated by an annotated circle. It is noticeable that the fragments representing helical conformations (S-W) are spaced much closer than those of extended conformations (A-I). Helical conformations can be well represented by a few similar states, while extended conformations are more versatile and require more representatives to capture the variability of strands in proteins.

**Figure 4:**
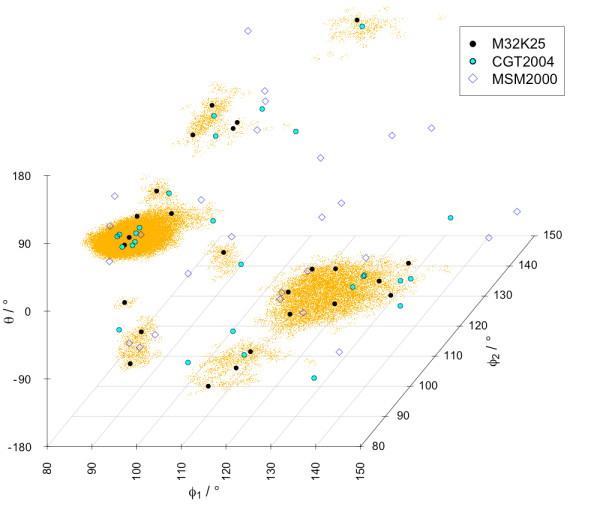
**Comparison of fragment location for Structural Alphabets of four *C*^*α *^atoms**. The fragment representatives for M32K25, MSM2000 [8] and CGT2004 [11] are plotted in conformational space [*ϕ*_1_, *ϕ*_2_, *θ*].

The *MinPts *parameter was used to fine-tune the extraction of Structural Alphabets to specific densities. We generated Reachability Plots for values of *MinPts *in the range 10-100. Structural Alphabets derived for *MinPts *values > 100 contain too few (< 14) fragments and already those derived for values > 60 show an unsatisfactory performance in reconstructing protein structures (global fit RMSDs 1.0-1.2 Å). Therefore, only results for *MinPts *values in the range 10-60 are reported here. Our alphabets are named according to their *MinPts *parameter and size, for example 'M32K25' for *MinPts *= 32 and k = 25. A comparison between the location of the fragments of three Structural Alphabets relative to the conformational attractors is given in Figure 4. The M32K25 set is compared with two alphabets from the literature of size 27 (CGT2004 [11]) and 28 (MSM2000 [8]), both composed by fragments of four *C*^*α*^. The centering of the M32K25 representatives at data clusters is clearly visible.

### Structural Alphabet performance assessment

Structural Alphabets capture the essence of conformational variability of the folded protein backbone in a small number of states. By extracting the states of highest density as representatives, we maximise the probability to match this state in any given structure. Therefore, in terms of a Structural Alphabet, a protein structure can be thought of as a sequence of conformational states and the representation of a protein structure can be reduced to a string of alphabet characters (structure string). This translation can be thought of as an encoding, achieved by matching the best fitting alphabet fragment to each position of the protein structure. This can be done for each position independently (local fit) allowing for non-exact fragment overlaps, or by searching for the sequence of fragments that comprehensively best approximate the geometry of the template structure (global fit) with exact fragment overlaps. In both cases the cRMSD between fitted and template structure is a measure for the error associated with the encoding. The two fit procedures exemplify also two extreme cases of the structure prediction problem: local and fold prediction. As the aim of this work is not to provide a new tool for structure prediction, these two fit assessments should not be interpreted as a measure of the predictive ability of the alphabets; further tests would be required to validate the alphabets in the context of fold prediction.

**Figure 5:**
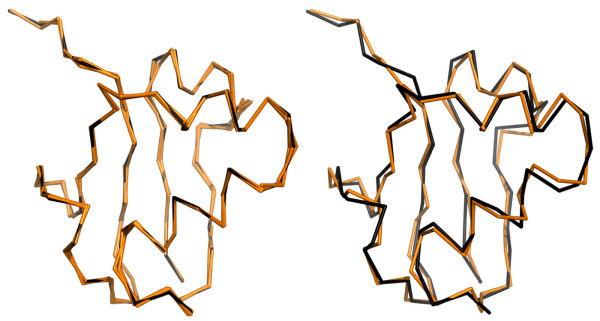
**An example of a typical local/global fit reconstruction**. Alignment of the template SCOP domain 1fm0d_(black) and the reconstructed structure (orange) for both, local (left) and global fit (right). Fit cRMSD values are 0.19 A (local) and 0.70 Å (global). The image was generated with PyMol [64].

The global fit error is generally higher than the local fit error, because it incorporates the deviation between the alphabet fragments and the template structure (like the local fit) and additionally the deviation arising from the neighbour fragment super-positioning. The global fit error is therefore a more stringent measure for the assessment of an alphabet's performance. An example of a typical local/global fit reconstruction is shown in Figure 5. The quality of each Structural Alphabet was assessed by its ability to approximate real protein structures both by local fit and global fit reconstruction [6] on a test set of 798 high quality protein structures. A summary of the assessment is reported in Table 2 and the complete table for *MinPts *10-60 is included as Additional File 1: Supplementary Table S1; for each set the alphabet size (k) is given as well as the median and inter quartile distance (IQD) of the cRMSD distribution. These are robust statistical results unaffected by the presence of outliers. The Structural Alphabets CGT2004 and MSM2000 from the literature are included for comparison. Since the fit results are equivalent whether done in the form of cRMSD or aRMSD (see Additional File 1: Table S2 and Additional File 1: Figure S4), we decided to employ the former for compatibility with previous studies [6,8,10,11].

**Table 2:** Performance assessment of Structural Alphabets in terms of the local and global fit quality.

	local fit		global fit		
alphabet	/Å	IQD/Å	/Å	IQD/Å	AIC/kbit
M12K31	0.238	0.108	0.885	0.214	-604
M16K28	0.225	0.077	0.770	0.213	-640
M20K27	0.216	0.060	0.733	0.138	-654
M24K23	0.227	0.066	0.783	0.144	-638
M28K21	0.233	0.064	0.823	0.132	-624
**M32K25**	**0.214**	**0.059**	**0.700**	**0.114**	**-668**
M36K20	0.240	0.062	0.855	0.125	-615
M40K20	0.244	0.065	0.867	0.138	-611
M44K17	0.257	0.072	0.955	0.147	-585
M48K17	0.255	0.071	0.918	0.156	-590
M52K21	0.242	0.072	0.772	0.168	-609
M56K17	0.253	0.070	0.909	0.170	-589
M60K16	0.259	0.072	0.952	0.146	-584

**CGT2004**	**0.218**	**0.062**	**0.666**	**0.150**	**-666**
MSM2000	0.286	0.124	0.946	0.414	-604
**MxK25GA**	**0.209**	**0.056**	**0.683**	**0.118**	**-676**

: median cRMSD, IQD: inter-quartile distance of cRMSD; AIC: Akaike Information Criterion. Alphabets are labelled with their MinPts parameter value (M) and alphabet size (K). The literature alphabets CGT2004 and MSM2000 are of size 27 and 28, respectively. Best performing alphabets are highlighted in bold font.

For all fragment sets the quality of the global fit is comparable to the experimental uncertainty in protein structure determination: the median cRMSD is in the range 0.70-1.00 Å with an IQD in the range 0.10-0.25 Å. The local fit results show that a representative fragment can be found for any local conformation with an average fit error in the range 0.2-0.3 Å.

**Figure 6:**
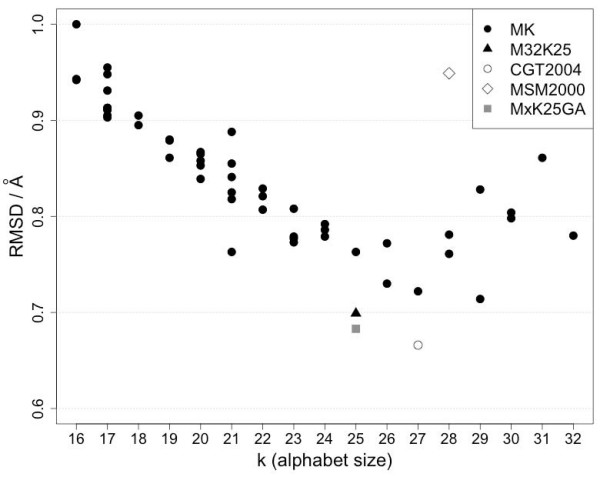
**Median global fit cRMSD against alphabet size (k)**. MK denotes the Structural Alphabets derived in this study. The test set comprises 798 high resolution protein structures. Symbols denote the alphabet type: (filled circle) the series of MxKy alphabets, (filled triangle) M32K25 alphabet, (empty circle) CGT2004 alphabet, (empty diamond) MSM2000 alphabet and (filled square) the alphabet resulting from the GA optimisation of all fragments contained in the MxKy series.

The size of a discrete state model is a trade-off between performance and complexity. A plot of the fit accuracy as a function of alphabet size is given in Figure 6. As predicted, the median cRMSD is generally decreasing when more representatives are included in the alphabet, but the trend is reversed beyond size 27. This is consistent with a density-based approach: as we increase the density cutoff, the fitness of the model increases by including still informative but rarer states until a global optimum is reached, after which any new states are not providing more information but add to the complexity.

Other groups have devised optimal alphabets of size 27 [11] and 28 [8]. While in those studies the identification of the optimal size was not done by fit performance, it is noteworthy that the range of optimal values is similar to the one identified by OPTICS.

**Figure 7:**
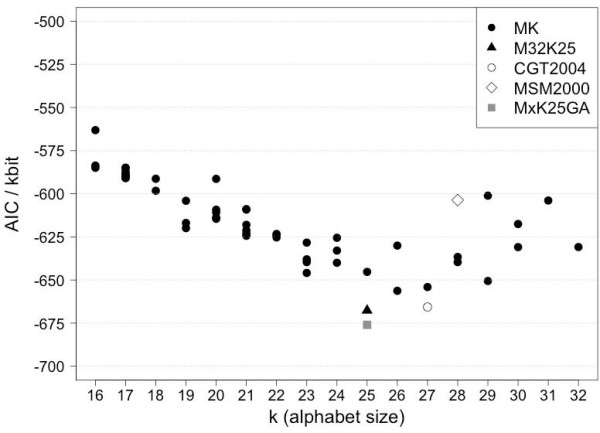
**Akaike's Information Content (AIC) against alphabet size (k)**. Alphabets and test set are identical to those in Figure 6. Symbols denote the alphabet type: (filled circle) the series of MxKy alphabets, (filled triangle) M32K25 alphabet, (empty circle) CGT2004 alphabet, (empty diamond) MSM2000 alphabet and (filled square) the alphabet resulting from the GA optimisation of all fragments contained in the MxKy series.

To correctly define the global optimum (best performing Structural Alphabet) we used Akaike's Information Content (AIC, see Methods) that allows comparison of fitness models having a different number of parameters. The results are reported in Figure 7. The trend is similar to the global fit plot in Figure 6 and confirms that the alphabet M32K25 derived for m = 32 (*MinPts*) and k = 25 (fragments) is performing best. In terms of the AIC, this alphabet (M32K25) is roughly equivalent to the alphabet CGT2004 [11] (k = 27). M32K25 is shown as a black triangle in Figure 6 and 7.

The robustness of the AIC test was confirmed by bootstrapping. The results of 10000 bootstraps are reported as error bars in the plot in Additional File 1: Supplementary Figure S3.

### Structural Alphabet M32K25

The location of each of the 25 fragments of the M32K25 alphabet in the conformational space is illustrated in Figure 2, where the fragments are labelled by capital letters according to their order in the Reachability Plot (see also top and middle panel of Figure 3). Angle values are reported in Table 3. The alphabet includes representative fragments for each of the populated conformations, sampling both dense and sparser regions. One can discern seven areas (note the periodic boundary of *θ*): (A-I), (J-L), (M-N), (O-R), (S-W), (X), (Y). These areas correspond to valleys in the Reachability Plot (Figure 3) that are separated by peaks of inter-cluster distances.

**Table 3:** Angle values of the Structural Alphabet M32K25.

fragment	*ϕ*_1_/°	*ϕ*_2_/°	*θ*/°
A	122.4	119.4	-164.2
B	129.8	135.6	-176.6
C	117.1	111.0	-142.2
D	118.4	126.9	-146.1
E	116.7	138.6	168.7
F	115.6	112.9	-117.9
G	135.3	118.6	-148.5
H	120.1	114.3	-90.7
I	133.6	117.1	-120.8
J	115.9	91.4	-134.6
K	119.7	90.4	-105.9
L	110.0	90.8	-158.8
M	110.0	100.8	177.0
N	90.1	138.2	19.6
O	92.4	91.2	-127.4
P	91.8	96.7	-104.8
Q	95.9	117.7	136.0
R	94.5	112.6	115.0
S	96.3	94.7	112.0
T	93.0	92.8	83.1
U	91.4	90.7	49.8
V	93.3	89.1	68.3
W	93.8	105.2	32.3
X	111.4	94.6	21.8
Y	89.0	95.1	-54.4

While all three angles (*ϕ*_1_, *ϕ*_2_, *θ*) are needed to fully describe the variety of conformations, the torsion angle *θ *provides most of the information. This is confirmed by the order of fragments in the Reachability Plot and illustrated by the selected fragments on the right panel of Figure 2: the order extended (A), loop (P), helical (U) and turn (Y) corresponds to a progressive decrease of the torsion angle *θ*.

**Figure 8:**
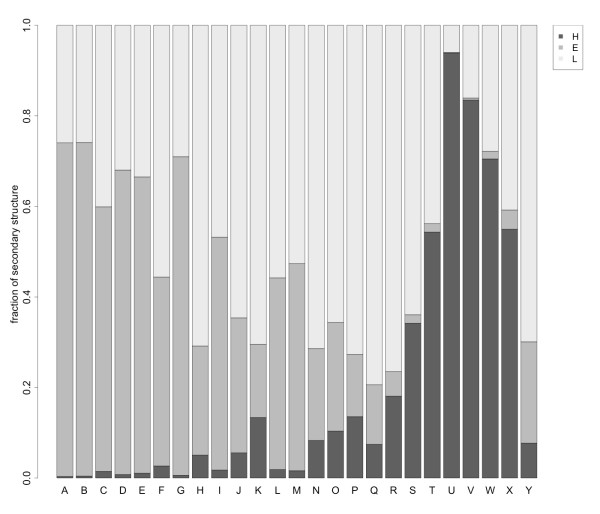
**Barplot of overall secondary structure contribution per letter of the Structural Alphabet M32K25**. The secondary structure attribution is based on the annotation from STRIDE on the second *C*^*α *^atom of each fragment.

This is also consistent with the secondary structure attribution of STRIDE [59] to the 798 proteins included in the test set: fragments (A-I) generally encode for strand-like regions, (J-L, M-N, O-R) for loops, (S-W, X) for helices and (Y) for turns. A detailed bar plot of the fraction of secondary structures associated with each letter of the alphabet is shown in Figure 8.

As previously reported [11], one should not expect a strict correspondence between the states in a Structural Alphabet and those obtained from secondary structure assignment for the same local structures. The possibility to fit some of the fragments to different structural environments is important to achieve high accuracy in protein reconstruction.

### Optimisation of combined alphabets

To investigate how the hierarchical cluster extraction scheme has biased the alphabet selection, we performed a separate extraction. A Genetic Algorithm optimisation was performed on the collection of all non-redundant fragments of all alphabets within the *MinPts *range 10-99. The optimisation was designed to select those 25 out of 106 fragments in the collection that yield the best local fit score on a set of 10 high quality structures.

The resulting alphabet (MxK25GA) was assessed by local fit, global fit and AIC performance (Table 2). The improvement in local fit (0.01 Å) and global fit (0.02 Å) is smaller than the variance in the corresponding fit processes and the AIC difference is also relatively small (8.3 kbit). The GA optimised set is indeed equivalent to the M32K25 alphabet, confirming the results of the hierarchical extraction procedure.

### Intrinsic flexibility of reconstructed proteins

The fit assessment is a simple and robust method to measure the accuracy of a Structural Alphabet in approximating real proteins, but it does not guarantee that all native features are correctly reproduced in the reconstructed structure [6].

While the M32K25 alphabet has satisfied the necessary requirement of reconstructing static structures within the experimental error, it is important to validate its ability to capture the intrinsic flexibility of proteins. This can be done by comparing the flexibility of the native and reconstructed structure: the dynamical properties should be unaffected by the discretisation imposed by the Structural Alphabet. An elegant and fast method to perform such an analysis is provided by the GNM [44,45], that has already been used in several structural studies [46] and proven to be a reliable approximation for the dynamic properties of proteins.

For our purpose, the cross-correlation of atomic fluctuations was derived by applying the GNM and compared in the native and reconstructed structures (see Methods). Both the atomic RMSF profiles and the cross-correlation matrices demonstrated the suitability of the M32K25 encoding. The reconstructed structures preserved the required native features: both the RMSF correlation (0.95 ± 0.04) and the matrix overlap (0.93 ± 0.02) are close to 1 for a large set of high quality structures. The former correlation is also higher than the one reported with B-factors [60], confirming that the encoding error is within the experimental uncertainty. Additionally the two indices are independent of the global and local fit quality measures (correlations < 0.3).

This is also an indirect test of the ability of a structural alphabet encoding to preserve native contacts. Indeed in this harmonic model the conformational freedom of each atom is a function of the number of neighbour interactions [44,45]. The preservation of native contacts is a necessary precondition to obtain similar flexibility profiles.

For comparison purposes, this test was performed also on the Structural Alphabets CGT2004 and MSM2000. Both alphabets performed as well as the M32K25 in preserving the native contacts and the intrinsic flexibility. The RMSF correlations were 0.95 ± 0.03 (CGT2004) and 0.92 ± 0.06 (MSM2000), while the matrix overlap was 0.93 ± 0.03 for both.

While with a different aim and procedure, a previous study [61] has also highlighted that pairwise contact specificity is greater in terms of structural letters than amino acids.

### Accuracy and robustness in encoding structural ensembles

To be able to capture the dynamical behaviour of a protein, a Structural Alphabet should be stable to small fluctuations on one side and it should reproduce transitions between different states on the other. Thus a first requisite of an alphabet is that, when used to encode different structures in a conformational ensemble, the variability of the letters is correlated with the flexibility of the position that they describe. This means that a position encoded by many different letters should also show large fluctuations. On the other hand, if a fragment is relatively rigid, only few letters should be sufficient to accurately represent it. We analysed the relationship between flexibility profiles and encoding variability by generating ensembles of 500 structures for a set of 24 proteins with the tCONCOORD method. This relies on a more accurate description of the molecule than the GNM approach used in the previous section. Moreover, it allows the breaking of native contacts, so that the generated ensembles contain also transitions between significantly different conformations. A generally good agreement has been found between tCONCOORD and Molecular Dynamics (MD) [49,53]. However the aim of the present test is to assess the performance of the alphabet in reproducing structural variability in ensembles, independently from the method used to generate them. Finally, since the increased computational cost of tCONCOORD prevented its application to the entire dataset used for the other alphabet assessments, a limited number of proteins had to be selected.

**Figure 9:**
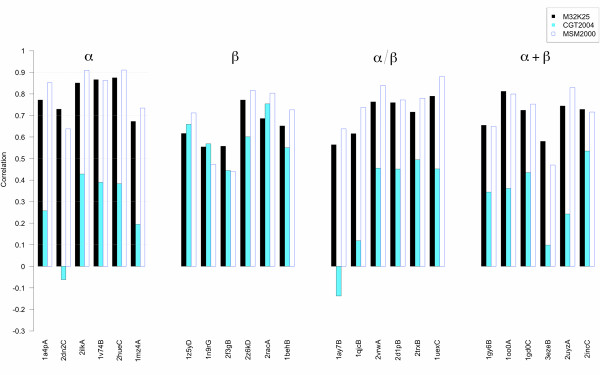
**Barplot of the Pearson correlation coefficients between RMSF and local-fit Shannon Entropy profiles**. The 24 proteins are ordered according to the SCOP class and, within a given class, to decreasing fraction of structured DSSP [55] elements. M32K25 values are reported in black, CGT2004 in light blue and MSM2000 as empty bars.

**Figure 10:**
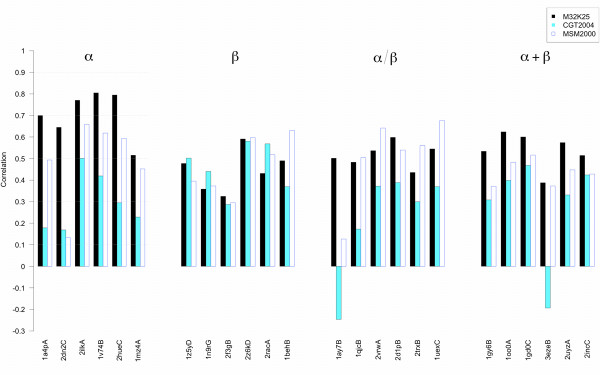
**Barplot of the Pearson correlation coefficients between RMSF and global-fit Shannon Entropy profiles**. See Figure 9 caption.

Correlations were calculated between the RMSFs of the fragments' geometries and the Shannon Entropies of their encodings (see Figure 9, Figure 10 and Table 4). Since the pure roto-translational motion of a given fragment does not contribute to a letter change in the encoding, it was eliminated in the RMSF calculation. Encodings using both local and global fit reconstructions were performed with the M32K25, CGT2004 and MSM2000 alphabets. From Figure 9 and 10, it is evident that the three alphabets have different performances. Moreover, the degree of the correlation does not mirror the accuracy of the reconstruction (see Table 5). When the structures are encoded by local fit (Figure 9), both the M32K25 and the MSM2000 alphabets show good correlation coefficients, which are below 0.6 only in four cases for M32K25 and in three cases for MSM2000. Smaller values are generally found for CGT2004, whose performance is comparable to the other two only for the *β *class. Conversely both M32K25 and MSM2000 better perform for proteins that contain *α*-helices (*α*, *α*/*β*, and *α*+*β*). Overall, M32K25 has the highest correlations in 7 cases, MSM2000 in 16 cases and CGT2004 in 1 case. However, differences between the M32K25 and MSM2000 values are often very small. When the global fit encoding is employed (Figure 10), the correlation values decrease for all alphabets: the reduced accuracy in the representation of the local structure required by a "seamless" reconstruction is a further source of letter variation, not necessarily related with the real flexibility of the fragment. The reduction in correlation affects more the MSM2000 alphabet, so that now M32K25 has the best performance in 15 cases, MSM2000 in 6 cases and CGT2004 in 3 cases.

**Table 4:** Correlation coefficient between fragment RMSF and encoding entropy.

		M32K25		CGT2004		MSM2000	
PDB ID	Chain	R(LF)	R(GF)	R(LF)	R(GF)	R(LF)	R(GF)
1a4p	A	0.772	0.700	0.258	0.178	0.852	0.493
2dn2	C	0.729	0.645	-0.062	0.169	0.638	0.133
2ilk	A	0.851	0.770	0.428	0.501	0.909	0.658
1v74	B	0.866	0.805	0.390	0.419	0.862	0.618
2hue	C	0.875	0.795	0.383	0.295	0.910	0.594
1mz4	A	0.672	0.515	0.195	0.228	0.734	0.452
1z5y	D	0.617	0.477	0.659	0.502	0.712	0.395
1n9r	G	0.554	0.358	0.569	0.440	0.473	0.373
2f3g	B	0.557	0.325	0.444	0.287	0.439	0.295
2z6k	D	0.772	0.591	0.601	0.580	0.816	0.597
2rac	A	0.686	0.431	0.754	0.568	0.804	0.518
1beh	B	0.652	0.490	0.550	0.369	0.726	0.630
1ay7	B	0.564	0.502	-0.137	-0.247	0.638	0.126
1qjc	B	0.616	0.483	0.119	0.172	0.738	0.504
2vrw	A	0.763	0.536	0.455	0.372	0.838	0.641
2d1p	B	0.759	0.598	0.451	0.388	0.772	0.539
2trx	B	0.716	0.435	0.494	0.299	0.780	0.560
1uex	C	0.790	0.545	0.452	0.369	0.881	0.676
1gy6	B	0.654	0.534	0.344	0.308	0.649	0.371
1oo0	A	0.812	0.624	0.362	0.399	0.799	0.483
1gd0	C	0.724	0.600	0.435	0.468	0.753	0.516
3eze	B	0.580	0.388	0.098	-0.194	0.470	0.373
2uyz	A	0.744	0.574	0.243	0.331	0.829	0.447
2inc	C	0.728	0.514	0.535	0.424	0.716	0.428

R(LF) and R(GF): Pearson correlation coefficients between fragment RMSF and Shannon Entropy of local fit (LF) and global fit (GF) encoding.

**Table 5:** Performance of Structural Alphabets in local and global fit reconstruction of tCONCOORD ensembles.

	M32K25 LF		M32K25 GF		CGT2004 LF		CGT2004 GF		MSM2000 LF		MSM2000 GF	
PDB ID	/Å	IQD/Å	/Å	IQD/Å	/Å	IQD/Å	/Å	IQD/Å	/Å	IQD/Å	/Å	IQD/Å
1a4p	0.225	0.014	0.664	0.037	0.207	0.014	0.575	0.046	0.261	0.012	1.005	0.034
2dn2	0.203	0.009	0.659	0.045	0.192	0.009	0.541	0.043	0.260	0.008	1.017	0.028
2ilk	0.261	0.013	0.771	0.046	0.237	0.013	0.673	0.053	0.286	0.011	1.024	0.025
1v74	0.218	0.016	0.653	0.046	0.209	0.015	0.559	0.049	0.280	0.014	1.018	0.025
2hue	0.250	0.017	0.700	0.045	0.230	0.016	0.622	0.051	0.291	0.014	1.001	0.029
1mz4	0.273	0.013	0.756	0.032	0.260	0.011	0.667	0.030	0.317	0.011	0.960	0.023
1z5y	0.308	0.021	0.852	0.085	0.307	0.016	0.873	0.082	0.356	0.015	0.940	0.055
1n9r	0.328	0.020	0.844	0.061	0.310	0.017	0.803	0.049	0.352	0.016	0.847	0.032
2f3g	0.294	0.011	0.808	0.026	0.299	0.011	0.798	0.025	0.338	0.009	0.854	0.017
2z6k	0.300	0.015	0.811	0.048	0.298	0.013	0.807	0.039	0.333	0.013	0.883	0.024
2rac	0.308	0.017	0.810	0.050	0.307	0.015	0.819	0.051	0.354	0.013	0.869	0.025
1beh	0.313	0.013	0.863	0.034	0.305	0.011	0.831	0.032	0.351	0.008	0.925	0.016
1ay7	0.248	0.014	0.654	0.030	0.239	0.015	0.601	0.045	0.307	0.013	0.963	0.025
1qjc	0.262	0.012	0.725	0.026	0.253	0.010	0.666	0.028	0.307	0.010	0.957	0.021
2vrw	0.268	0.013	0.721	0.028	0.264	0.013	0.688	0.031	0.311	0.012	0.912	0.015
2d1p	0.275	0.018	0.749	0.032	0.269	0.016	0.718	0.032	0.331	0.014	0.913	0.023
2trx	0.305	0.016	0.763	0.032	0.289	0.015	0.703	0.035	0.328	0.011	0.929	0.023
1uex	0.279	0.012	0.763	0.029	0.265	0.010	0.722	0.028	0.318	0.014	0.975	0.018
1gy6	0.271	0.023	0.807	0.094	0.259	0.019	0.788	0.096	0.328	0.020	0.955	0.059
1oo0	0.279	0.020	0.797	0.071	0.263	0.018	0.739	0.072	0.320	0.016	0.969	0.039
1gd0	0.286	0.017	0.819	0.046	0.266	0.015	0.757	0.053	0.337	0.014	0.959	0.038
3eze	0.284	0.017	0.735	0.036	0.269	0.016	0.694	0.047	0.314	0.015	0.921	0.042
2uyz	0.268	0.015	0.745	0.044	0.268	0.013	0.745	0.045	0.313	0.011	0.946	0.022
2inc	0.268	0.016	0.728	0.052	0.285	0.016	0.744	0.049	0.324	0.014	0.878	0.035

: median cRMSD, IQD: inter-quartile distance of cRMSD. Values from the M32K25, CGT2004 and MSM2000 alphabets are calculated by local fit (LF) and global fit (GF) encoding.

**Figure 11:**
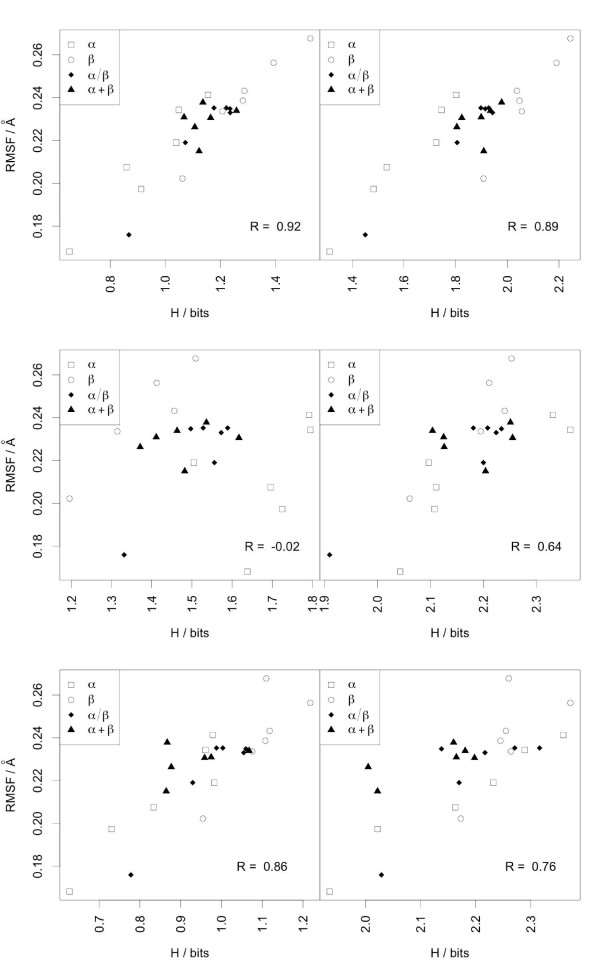
**Scatterplot of the average protein RMSF against the average Shannon entropy**. Average RMSF values (in Å) calculated over all the residues of each of the 24 proteins are reported against the average Shannon Entropy (in bits) for the M32K25 (upper panel), the CGT2004 (middle panel) and the MSM2000 (lower panel) alphabets. Left and right scatterplots contain Shannon Entropies from the local and the global fit reconstructions, respectively. Empty squares are used for *α*-class proteins, empty circles for *β*, filled diamonds for *α*/*β *and filled triangles for *α*+*β*.

When correlations are calculated per protein rather than per residue, i.e. by comparing the average protein RMSF with the average entropy (Figure 11), good results are again obtained for M32K25 and MSM2000 with correlations greater than 0.7. In this case the M32K25 alphabet best performs independently from the type of reconstruction. Correlation values are much smaller for the CGT2004 alphabet, in particular if the entropy from local fit encoding is considered.

## Discussion

We have derived the structural alphabet M32K25 from the conformational attractors of protein structures. The intention of our approach was to devise a simple and generic description of local conformations that is readily amenable to visualisation and computational analysis. A solution was found in the conformational space spanned by three internal angles corresponding to the fragment's degrees of freedom.

The OPTICS algorithm provided two important functions for the analysis of the data space. Firstly, cluster representatives were extracted in the order of decreasing density, which is equivalent with decreasing importance for encoding. Secondly, the unique data ordering corresponds to a minimum distance path, providing a gradual inter-conversion among the states. Therefore, despite the mutual independence between fragments, the resulting Structural Alphabet includes important connective fragments to allow for a smooth protein reconstruction. Connectivity is partially implied by the overlap of neighboured fragments in the original structures: the *ϕ*_2 _angle of a fragment in a given structure is identical to the *ϕ*_1 _angle of the next C-terminal fragment. The addition of a structural alphabet fragment to a growing reconstruction model adds one unconstrained atom (while three atoms overlap with the previous fragment) and two unconstrained angles *ϕ*_2 _and *θ *(while *ϕ*_1 _overlaps with *ϕ*_2 _of the previous fragment).

The OPTICS algorithm has been recently applied for sequence clustering [62], but to the knowledge of the authors, this is the first use in Structural Bioinformatics and it suggests a general suitability of density-based approaches for protein structure analysis.

A further objective of this study was to minimise the number of free parameters in design and analysis. Excluding the descriptive conformational parameters (*ϕ*_1_, *ϕ*_2_, *θ*), only the *MinPts *parameter influenced the set of representative fragments. We explored the range of suitable values for *MinPts *and selected the most informative alphabet. The high entropy of the M32K25 alphabet allows for protein reconstruction with an error comparable to that of structure resolution techniques. Theoretical studies on small libraries of local structures [6,10] predict the global fit accuracy for an alphabet of this size to 0.60 Å in agreement with our results (0.70 ± 0.11 Å).

A comparison can be drawn directly only to other alphabets composed by fragments of the same type, i.e. of length four *C*^*α *^atoms. Previous Structural Alphabets used a fragment representation in the form of a set of Cartesian coordinates (MSM2000 [8]) or of a four-vector descriptor composed of three not-consecutive *C*^*α*^-*C*^*α *^distances and the projection of the fourth atom onto the plane formed by the other three (CGT2004 [11]). Our angular representation has the advantage of being an internal metric that is independent of the molecular orientation, as for CGT2004, but with only three parameters. The other alphabets include collections of 27 fragments (CGT2004) and 28 fragments(MSM2000), while our best alphabet M32K25 includes 25 states. The performance as measured by the global fit (shown in Table 2) is 0.70 ± 0.11 for M32K25, 0.67 ± 0.15 for CGT2004 and 0.95 ± 0.41 for MSM2000, indicating that the M32K25 alphabet achieves similar or better performance with only 25 states.

But the main difference between the M32K25 alphabet and other Structural Alphabets is its stringency in the representation of high density states as shown by the fragment locations in Figure 4: other approaches were equally successful in describing only some of the attractors. The efficiency in our extraction was achieved by including a minimal number (three) of the most informative (angular) degrees of freedom to describe each fragment and by analysing selected high quality structures.

Associating physico-chemical properties to the M32K25 fragments automatically maps these properties onto the most important conformational states. The simplicity of this mapping together with the option to visualise the map should be useful for protein structure analysis and design.

The main advantage of a density-based selection is the ability to directly capture the most highly populated conformations; these have also a higher chance to be sampled during protein dynamics. Borrowing Anfinsen's 'thermodynamic hypothesis' one may speculate that the alphabet fragments correspond to low energy conformations, because proteins can be reconstructed using solely these fragments.

We investigated the suitability of the M32K25 alphabet and its associated mapping in the analysis of conformational ensembles of protein structures.

A precondition for this type of conformational analysis is that the alphabet encoding can correctly preserve the intrinsic flexibility of a protein structure. This was demonstrated for the M32K25 by an assessment based on GNM calculations: the native contacts and the flexibility calculated with this harmonic model were completely preserved in the reconstructed structures. An extension of the GNM calculations to structures reconstructed with the CGT2004 and MSM2000 alphabets also suggests that this fidelity is a general property of structural alphabets, but not directly correlated to the accuracy in fit reconstruction. A previous study [61] has also demonstrated that the CGT2004 alphabet has more specificity than the amino acid code in capturing inter-residue contacts in protein complexes.

The ability to capture the dynamical behaviour of a protein was tested by encoding the different structures in conformational ensembles generated by tCONCOORD. We measured the accuracy and robustness of M32K25, CTG2004 and MSM2000 alphabets by the correlation between the Shannon Entropy of the encoded ensemble and its fragment flexibility in terms of RMSF.

Correlations are generally higher for local than global fit encoding, because optimal global reconstruction is achieved at expense of local accuracy. This suggests the importance of designing strategies to estimate the weight of this inaccuracy in the encoding. All three alphabets have comparable results for *β*-class proteins, but the performances are significantly better for M32K25 and MSM2000 in the other SCOP classes. Where the former has the best performance in global fit and the latter in local fit (see Table 4 for details).

M32K25 is the more efficient in capturing the average flexibility per protein (see Figure 11).

The performance difference between the structural alphabets can be explained in terms of robustness. A set of representatives that efficiently samples the conformational space with low redundancy will be less affected by small fluctuations, while a set that contains groups of relatively similar fragments describing the same state will tend to overestimate the conformational difference. This is a possible explanation for the performance of CGT2004 for *α*-helix containing proteins: the alphabets includes a group of closely located fragments in the α region of the [*ϕ*_1_, *ϕ*_2_, *θ*] space (see Figure 4). On the contrary a set of well spaced fragments does not imply an accurate encoding. The good performance of MSM2000 does not correspond to a good accuracy in the reconstruction (see Table 5).

The fragment composition of a structural alphabet is dependent on the type of strategy employed to select conformational representatives. This can affect the overall encoding stability. Both M32K25 and MSM2000 were derived by indirectly maximizing the geometrical diversity, while CGT2004 was optimized for statistical representativity. The former strategy provides a clear advantage in terms of encoding stability at the expense of a minor (M32K25) to significant (MSM2000) decrease in the encoding accuracy, while the reverse is true for the CGT2004 set: the inclusion of statistically significant but geometrically similar helical conformations can decrease the stability but provides a very accurate description of linear, kinked and curved helices [11]. This limitation in encoding stability could be overcome by considering the states as not strictly independent and consequently by either weighting their contributions according to their geometrical dissimilarity or by constructing a suitable substitution matrix. A successful example of the latter approach has been already used in the context of 3D structural alignment [21], where the performance of string-based structural comparison was increased allowing non-exact matches by means of a structural alphabet substitution matrix.

We do not aim to provide an alternative framework for structure prediction, but a novel tool for studies of protein structures and their dynamics. The addition of this newly designed assessment to the ones previously proposed in the literature is in line with the purpose of our alphabet. The density-based M32K25 alphabet has proven to be accurate for protein reconstruction and stable for ensemble encoding. The combination of these features suggests that M32K25 is specifically suitable for studies of protein dynamics.

## Conclusions

The density-based Structural Alphabet provides a two-fold advantage: ensembles of protein structures can be encoded with high accuracy and sufficient robustness to correctly describe local flexibility.

Future developments may involve the employment of this Structural Alphabet to analyse and annotate structure ensembles from Molecular Simulations to easily map molecular motions onto the fragment space. The attractors can act as a guide to classify dynamics features and to compare protein families or different energetic states of the same protein. This can help in understanding, for example, binding specificity to multiple partners or conserved biological mechanisms.

## Authors' contributions

AP and JK jointly conceived the study and implemented the code. AP and AF designed and carried out the data analysis. AP, AF and JK wrote the manuscript and approved the final version.

## Supplementary Material

Additional file 1 **Supplementary tables and figures**. The file contains Figure S1: Flow-chart of the OPTICS algorithm; Figure S2: Projection of ASTRAL SCOP 10 fragments into the conformational space of the internal angles (*ϕ*_1_, *ϕ*_2_, *θ*); Figure S3: Akaike's Information Content (AIC) against alphabet size (k) with bootstrapping; Figure S4: Comparison of the aRMSD and cRMSD distribution of matched fragments for the local fit of the protein test set; Table S1: Performance assessment of Structural Alphabets in terms of the local and global fit quality; Table S2: Fragment statistics of alphabet M32K25 for local fit. Click here for file
