# Supplementary material for: Structural alphabets derived from attractors in conformational space
Source: BMC Bioinformatics. 2010 Feb 20;11:97. doi: 10.1186/1471-2105-11-97 (PMC2838871; doi:10.1186/1471-2105-11-97)
Supplement: Additional file 1 — Supplementary tables and figures. The file contains Figure S1: Flow-chart of the OPTICS algorithm; Figure S2: Projection of ASTRAL SCOP 10 fragments into the conformational space of the internal angles (ϕ1, ϕ2, θ); Figure S3: Akaike's Information Content (AIC) against alphabet size (k) with bootstrapping; Figure S4: Comparison of the aRMSD and cRMSD distribution of matched fragments for the local fit of the protein test set; Table S1: Performance assessment of Structural Alphabets in terms of the local and global fit quality; Table S2: Fragment statistics of alphabet M32K25 for local fit. [file 1471-2105-11-97-S1.PDF]

Supplementary Figures

Figure S1: Flow-chart of the OPTICS algorithm

The data are provided as an unsorted list. See Manuscript for the definition of CD (Core Distance), ϵ, $MinPts$. Distance $d(i,j)$ is the Euclidian distance between point i and j in the data space. RD_j is defined as the maximum between $d(i,j)$ and CD_i.

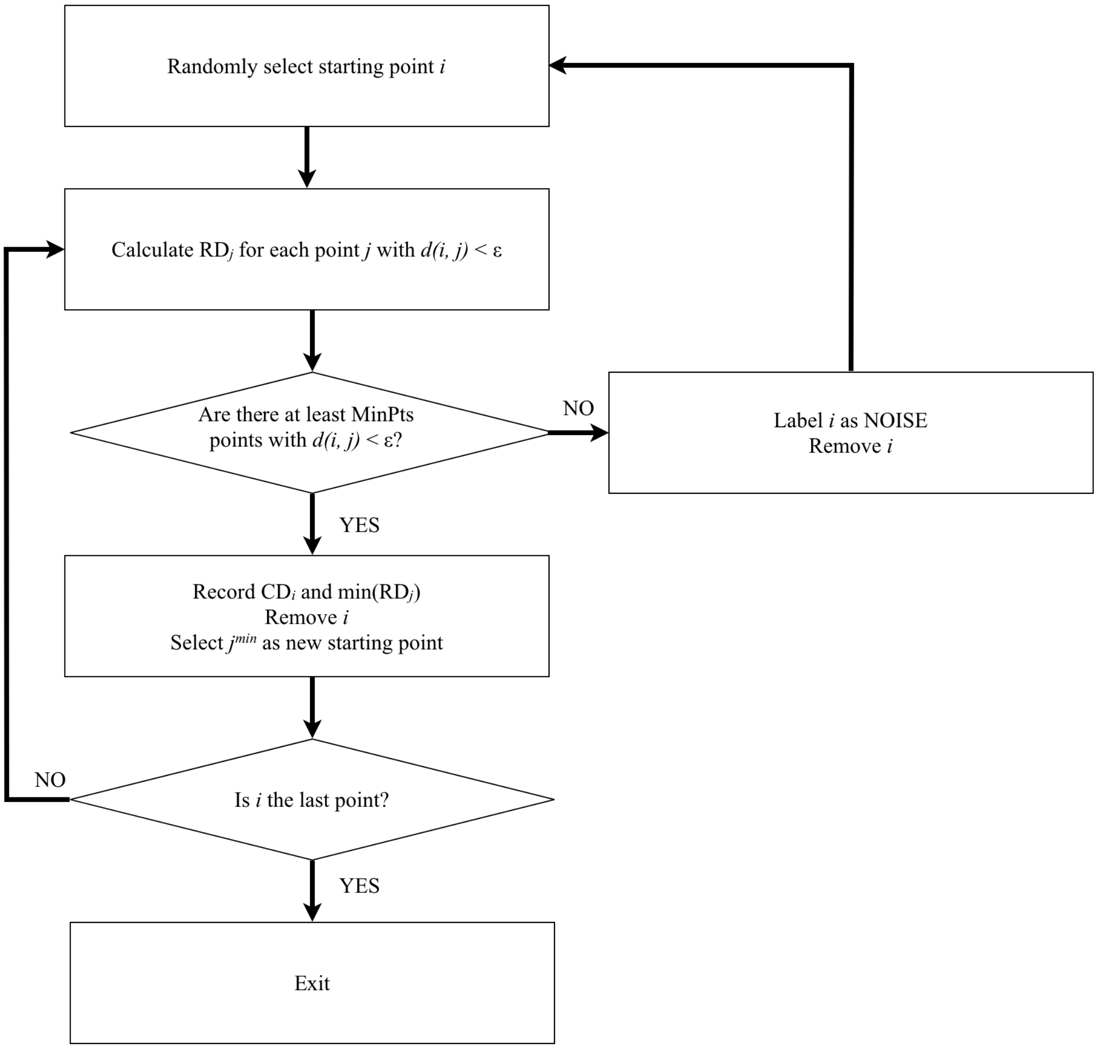

Figure S2: Projection of ASTRAL SCOP 10 fragments into the conformational space of the internal angles (ϕ_1,ϕ_2,θ)

Subset with resolution smaller than 1.75 Å (a), 2.00 Å (b), 2.25 Å (c), 2.50 Å (d) are reported in the figure. Fragments are colored according to the density in a cubic grid with 2° resolution: fragments in regions with density values in the top 30% of the range are colored in red, in the top 20% in orange, in the top 10% in yellow and in the top 5% in green. The dataset employed in the OPTICS analysis corresponds to the 2.00 Å subset of ASTRAL SCOP 10.

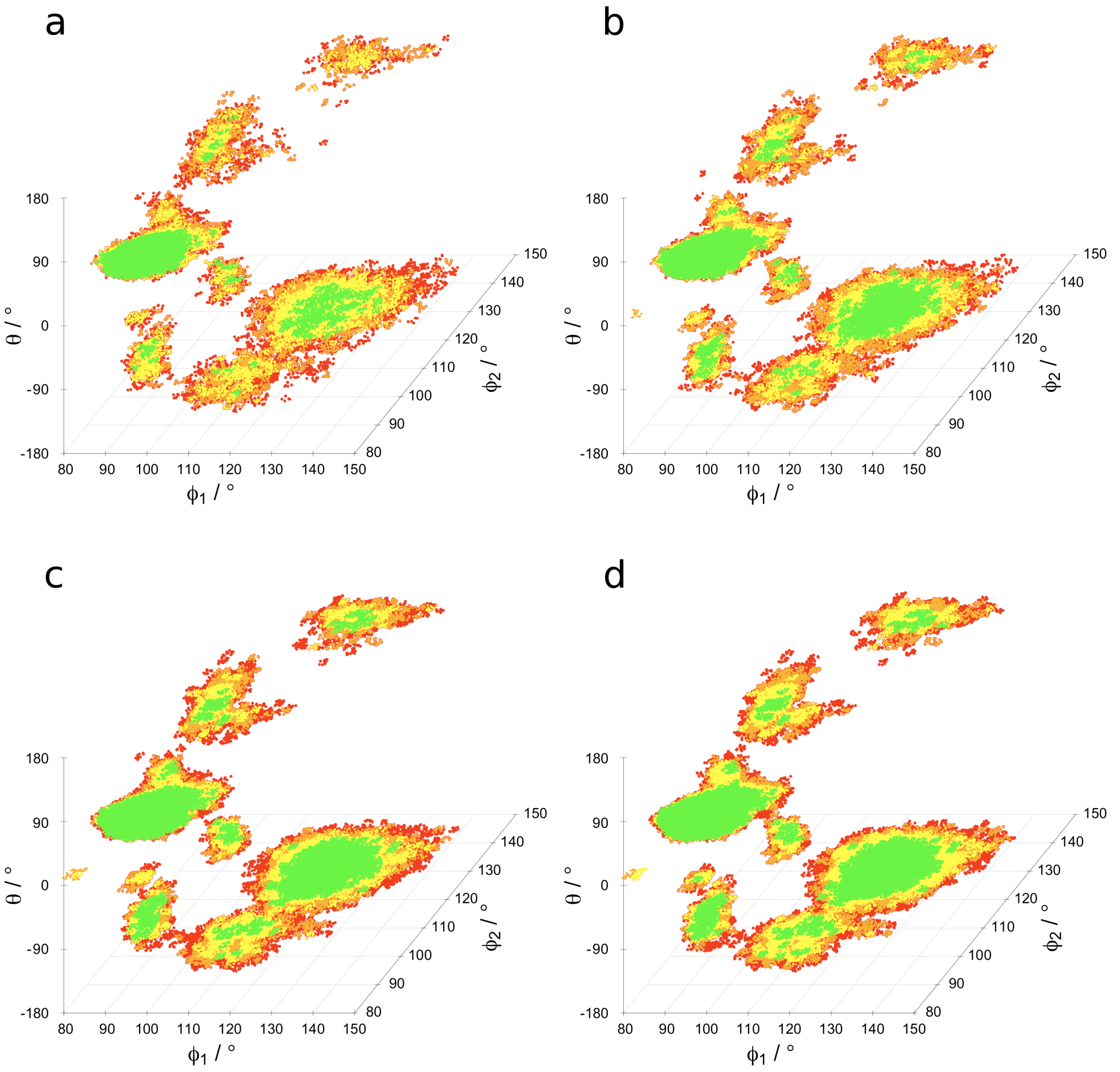

Figure S3: Akaike's Information Content (AIC) against alphabet size (k) with bootstrapping

MK denotes the Structural Alphabets derived in this study. The test set comprises 798 high resolution protein structures. Symbols denote the alphabet type: (filled circle) the series of MxKy alphabets, (filled triangle) M32K25 alphabet, (empty circle) CGT2004 alphabet, (empty diamond) MSM2000 alphabet and (filled square) the alphabet resulting from the GA optimisation of all fragments contained in the MxKy series. Best performing MxKy per size are highlighted in blue. The error bar represent the IQD calculated by bootstrapping.

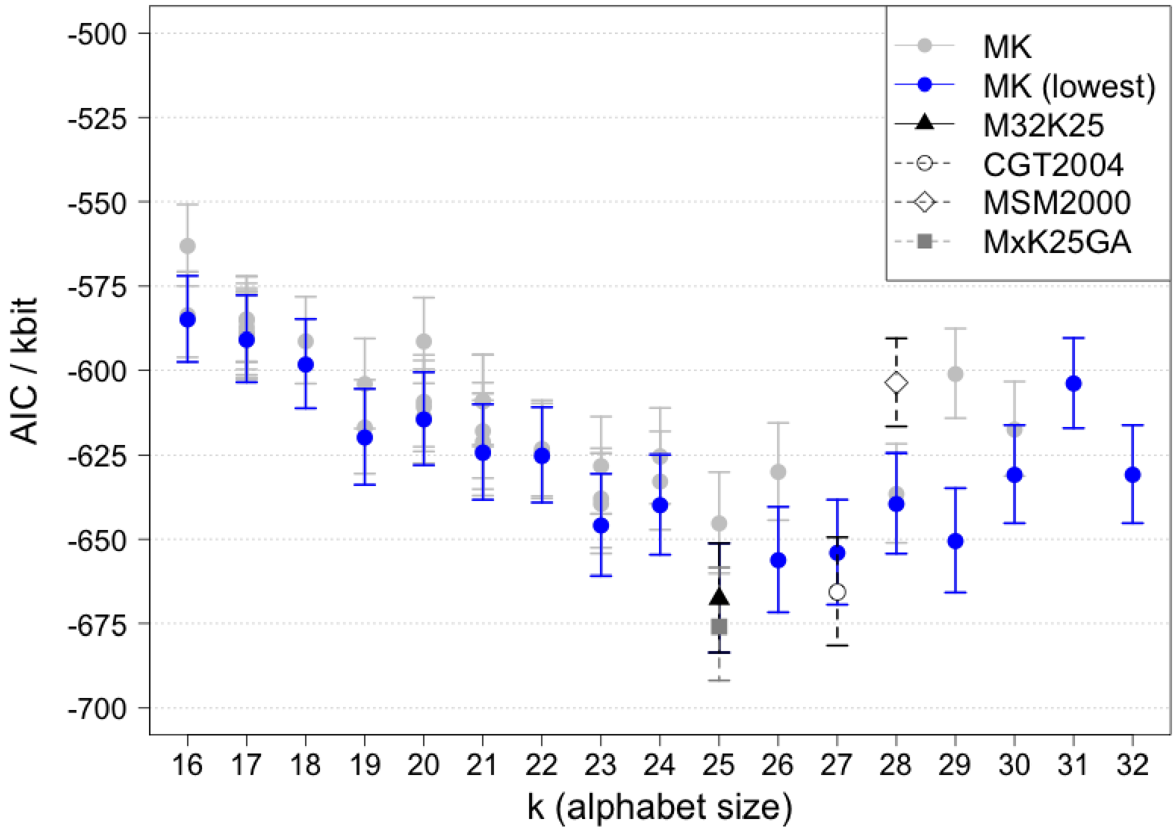

Figure S4: Comparison of the aRMSD and cRMSD distribution of matched fragments for the local fit of the protein test set

aRMSD (grey) and cRMSD (hatched) distributions were normalised by their estimated 'location' and 'scale' parameters, after extreme value distribution fit ('fgev' function of R-project package 'evd').

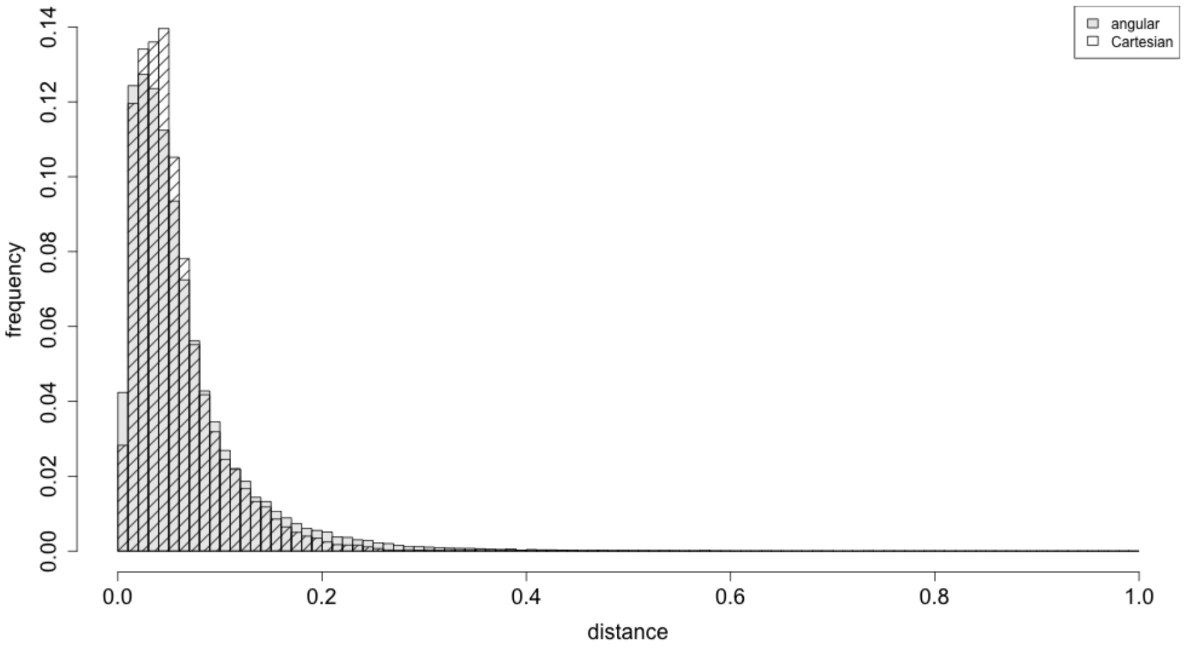

Supplementary Tables

Table S1: Performance assessment of Structural Alphabets in terms of the local and global fit quality

$\widetilde{x}$: median cRMSD, IQD : inter quartile distance of cRMSD; AIC : Akaike Information Criterion. Alphabets are labelled with their *MinPts* parameter value (M) and alphabet size (K).

		local fit		global fit		
alphabet	k	$\widetilde{x}$ / Å	IQD / Å	$\widetilde{x}$ / Å	IQD / Å	AIC / kbit
M10K32	32	0.223	0.074	0.791	0.190	-631
M11K30	30	0.231	0.083	0.816	0.215	-618
M12K31	31	0.238	0.108	0.885	0.214	-604
M13K29	29	0.243	0.088	0.846	0.228	-601
M14K30	30	0.232	0.086	0.825	0.223	-631
M15K21	21	0.244	0.075	0.899	0.163	-609
M16K28	28	0.225	0.077	0.770	0.213	-640
M17K26	26	0.230	0.075	0.776	0.201	-630
M18K28	28	0.229	0.070	0.790	0.137	-637
M19K24	24	0.229	0.069	0.795	0.125	-640
M20K27	27	0.216	0.060	0.733	0.138	-654
M21K29	29	0.220	0.061	0.716	0.147	-651
M22K24	24	0.230	0.063	0.783	0.159	-626
M23K25	25	0.225	0.061	0.770	0.128	-645
M24K23	23	0.227	0.066	0.783	0.144	-638
M25K26	26	0.221	0.061	0.730	0.130	-656
M26K23	23	0.220	0.063	0.776	0.138	-646
M27K23	23	0.227	0.065	0.781	0.131	-640
M28K21	21	0.233	0.064	0.823	0.132	-624
M29K23	23	0.229	0.063	0.809	0.133	-628
M30K21	21	0.233	0.063	0.827	0.137	-623
M31K22	22	0.232	0.065	0.830	0.149	-624
M32K25	25	0.214	0.059	0.700	0.114	-668
M33K22	22	0.232	0.066	0.813	0.155	-623
M34K24	24	0.232	0.065	0.787	0.144	-633
M35K22	22	0.234	0.064	0.832	0.124	-625
M36K20	20	0.240	0.062	0.855	0.125	-615
M37K19	19	0.240	0.063	0.879	0.116	-617
M38K21	21	0.234	0.064	0.843	0.133	-621
M39K19	19	0.238	0.065	0.862	0.125	-620
M40K20	20	0.244	0.065	0.867	0.138	-611
M41K19	19	0.246	0.068	0.885	0.139	-604
M42K20	20	0.240	0.068	0.869	0.144	-609
M43K21	21	0.239	0.067	0.853	0.132	-618
M44K17	17	0.257	0.072	0.955	0.147	-585
M45K20	20	0.256	0.074	0.847	0.148	-591
M46K17	17	0.257	0.070	0.960	0.127	-585
M47K20	20	0.238	0.066	0.860	0.140	-614

continued

M48K17	17	0.255	0.071	0.918	0.156	-590
M49K16	16	0.258	0.072	0.945	0.123	-585
M50K17	17	0.255	0.069	0.906	0.151	-590
M51K16	16	0.268	0.081	1.007	0.201	-563
M52K21	21	0.242	0.072	0.772	0.168	-609
M53K18	18	0.250	0.068	0.896	0.144	-598
M54K17	17	0.253	0.073	0.905	0.157	-591
M55K17	17	0.254	0.071	0.917	0.148	-590
M56K17	17	0.253	0.070	0.909	0.170	-589
M57K18	18	0.250	0.075	0.908	0.165	-591
M58K17	17	0.255	0.073	0.935	0.157	-587
M59K17	17	0.252	0.075	0.915	0.155	-591
M60K16	16	0.259	0.072	0.952	0.146	-584
CGT2004	27	0.218	0.062	0.666	0.150	-666
MSM2000	28	0.286	0.124	0.946	0.414	-604
MxK25GA	25	0.209	0.056	0.683	0.118	-676

Table S2: Fragment statistics of the M32K25 alphabet for the local fit of the protein test set.

Given are the number N of occurrences, the median ($\widetilde{x}$) and inter quantile distance (IQD) of the matched fragment angles, their aRMSD values and cRMSD values.

fragment		ϕ_1		ϕ_2		θ		aRMSD		cRMSD	
	N	$\widetilde{x}$ / °	IQD / °	$\widetilde{x}$ / °	IQD / °	$\widetilde{x}$ / °	IQD / °	$\widetilde{x}$ / °	IQD / °	$\widetilde{x}$ / Å	IQD / Å
A	5277	121	10	120	8	-166	9	10	5	0.18	0.06
B	4618	130	12	134	8	-175	10	13	7	0.19	0.07
C	2999	117	9	112	8	-153	7	11	5	0.18	0.05
D	4628	115	12	127	9	-159	25	14	9	0.22	0.09
E	5010	115	11	137	10	169	6	17	13	0.25	0.12
F	3532	116	10	115	10	-121	13	12	7	0.19	0.06
G	4346	137	10	120	13	-153	31	13	9	0.20	0.08
H	5643	127	17	114	11	-101	42	20	25	0.30	0.19
I	4928	135	11	117	12	-126	8	15	11	0.22	0.09
J	3042	120	16	92	6	-128	3	13	8	0.22	0.08
K	4020	126	16	92	5	-117	20	15	14	0.24	0.13
L	2496	113	13	94	6	-154	4	12	7	0.22	0.08
M	4094	109	13	100	11	176	20	15	10	0.27	0.13
N	4846	98	10	135	13	24	3	29	23	0.40	0.19
O	1510	94	7	92	6	-123	12	10	6	0.18	0.08
P	1400	92	6	97	7	-103	5	9	8	0.16	0.09
Q	4179	96	8	119	16	119	2	16	13	0.27	0.16
R	4973	96	7	123	16	111	11	20	22	0.31	0.20
S	2323	97	7	95	7	105	20	13	11	0.23	0.12
T	2373	94	9	98	15	85	18	15	16	0.29	0.18
U	39698	91	3	92	3	59	.4	5	4	0.09	0.05
V	6013	91	5	90	5	75	.6	8	5	0.15	0.05
W	4557	94	7	103	8	46	5	10	6	0.19	0.07
X	3724	113	14	94	6	-18	16	20	18	0.34	0.20
Y	1022	95	16	94	6	-37	2	17	22	0.33	0.30
